# Assessment of the Anthropogenic Impact and Distribution of Potentially Toxic and Rare Earth Elements in Lake Sediments from North-Eastern Romania

**DOI:** 10.3390/toxics10050242

**Published:** 2022-05-10

**Authors:** Laurentiu Valentin Soroaga, Cornelia Amarandei, Alina Giorgiana Negru, Romeo Iulian Olariu, Cecilia Arsene

**Affiliations:** 1 Faculty of Chemistry, “Alexandru Ioan Cuza” University of Iasi, 11 Carol I, 700506 Iasi, Romania; laurentiu.soroaga@uaic.ro (L.V.S.); cornelia.amarandei@uaic.ro (C.A.); 2 ICI-CERNESIM, “Alexandru Ioan Cuza” University of Iasi, 11 Carol I, 700506 Iasi, Romania; alina.negru@uaic.ro; 3 RECENT-AIR, “Alexandru Ioan Cuza” University of Iasi, 11 Carol I, 700506 Iasi, Romania

**Keywords:** elements, sediment, spatial distribution, pollution assessment, ICP-MS, Podu Iloaiei Dam Lake

## Abstract

Chemical analysis was performed on sediment samples collected in two sampling sessions (July and October) from Podu Iloaiei Dam Lake, one of the most important water resources used for aquaculture in north-eastern Romania. The concentration of 15 trace elements (TEs), 8 refractory elements (REs), and 15 rare earth elements (REEs)—determined using inductively coupled plasma mass spectrometry—showed variability largely dependent of the sampling points and collection time. Manganese was the most abundant TE, V and Cr were the most abundant REs, while Ce was one of the most abundant REEs. The cerium negative anomaly and Gd positive anomaly were observed in the Chondrite-normalized distributions. In October, the Ce anomaly showed significant negative correlation with Mn, emphasizing the water body oxidation potential. The identified positive Gd anomaly was most likely associated with the use of Gd-chelating agents in magnetic resonance imaging in Iasi, the largest medical hub in north-eastern Romania. Principal component analysis extracted three factors explaining 96.0% of the observed variance, i.e., rock weathering, leaching from soil surface, contributions from urban stormwater and atmospheric deposition (50.9%), pedological contributions (23.7%), and mixed anthropogenic sources (e.g., traffic, waste discharge, agricultural activities; 21.4%). The evaluation of pollution indices highlighted low and moderate degrees of contamination for most of the elements and a considerable degree of contamination for Cd. Assigned Cd sources included fertilizers and pesticides used in the near agricultural areas or the high traffic road located near the lake. Since contamination of aquatic ecosystems with harmful elements is a human health concern, further monitoring of specific vectors in the food chain of the investigated dam lake will be of the utmost importance.

## 1. Introduction

The nature of element sources in sediments, combined with their different mobility, persistence, and toxicity, are critical factors in the assessment of their environmental impact [1]. Determining the concentration and distribution of elements provides an accessible method to evaluate anthropogenic impact on lake sediments and may represent an extent of biological stress caused by contaminants [2].

In urbanized areas, several heavy metals at trace levels are assigned as major pollutants. The direct discharge of industrial effluents, domestic wastewater into the environment, and use of agricultural chemicals has led to elevated heavy metal concentrations in urban soils and surface water bodies [3,4,5]. In lake sediments, the accumulation of heavy metals may negatively impact the status of the biota. Heavy metal accumulation in fish tissue through the food chain may pose a health risk to humans [6,7].

The extensive use of relatively new technologies is a major source of rare earth elements (REEs) in the environment, and the emission of REEs into natural waters is increasing [8]. The most important input of REEs in the environment is comprised by the fertilizers used in agriculture [9,10], intensive use in the industry, specific medical applications [9,11], and dry deposition of aerosol particles [8]. The REEs are characterized by very similar physicochemical behaviors [12,13], are very good tracers of chemical [14,15,16] and geochemical [9,16,17] processes, and may represent a new class of micropollutants that are not fully understood and could impact the environment.

Although, in aqueous environments, the REEs chemistry involves almost exclusively the trivalent oxidation state, stable compounds formed by Ce^4+^ and Eu^2+^ are of high interest [13,18], as they may fractionate from the trivalent state REEs, according to the water redox potential [12,13,19]. In well-oxygenated aqueous environments, bacterial activity mediated oxidation of Ce^3+^ to Ce^4+^ may occur [20], with the tetravalent state associated mainly with the water insoluble solid phase [19]. In this case, some sort of Ce depletion is observed in the aqueous phase, leading to a different distribution than the normal crust abundances of REEs, a process known as negative Ce anomaly [12,18]. Both the positive and negative Ce anomaly (Ce/Ce*) can be used for the evaluation of the redox conditions of aquatic environments [12,13,21]. Gadolinium is another REE susceptible to anomalies, and its positive anomaly (Gd/Gd*) is most likely associated with anthropogenic sources related to the use of Gd-chelating agents in magnetic resonance imaging (MRI) [11,22,23].

The spatial distribution characteristics of metals in sediment may indirectly give an indication of the pollution sources localization. Multivariate statistics techniques (e.g., principal component analysis, correlation analysis) are powerful tools for pollution source assessments [4,24]. These techniques were applied for the investigation of pollutants in soil, sediment [25,26,27,28], and natural waters [29,30].

Sediment contamination across a wide range of environments is reported in several studies [1,2,3,31]. Contamination with various elements is often assessed using approaches such as contamination factor (CF), pollution load index (PLI), geoaccumulation index (*I_geo_*), and enrichment factor (EF) [32,33,34,35]. While PLI standardizes the contribution from the elements and is used to indicate bioavailability [33], EF allows to identify natural variations of a given element in order to detect even a very small anthropogenic impact and to find out the extent of the element contaminants to be evaluated in toxicological studies [36,37]. In the estimation of the EF, geochemical elements present in large quantities (e.g., Mn) [1,34] are usually used as reference elements to determine anthropogenic enrichment. Moreover, freshwater sediment quality guidelines (SQGs) are useful in assessing the sediment quality and the potential of toxic elements to cause negative effects on aquatic organisms [38,39]. Consensus-based SQGs, namely the Threshold Effect Concentration (TEC) and Probable Effect Concentration (PEC) [1,3,39], can be used for assessing element effects on the environment. No adverse effects of the contaminant on organisms are expected to occur at concentration levels below TEC. Potential toxic effects may or may not occur for values greater than or equal to TEC but lower than PEC. At concentrations above PEC, probable toxic effects of contaminants would be very likely to occur [39,40].

To our knowledge, limited information on lake sediment quality in north-eastern Romania is available. Strungaru et al. [6] studied the potential contamination with Cu, Cd, Pb, Cr, and Ni of water and sediment in the Stanca-Costesti freshwater reservoir from north-eastern Romania. Although the information is valuable, the number of investigated contaminants is scarce and the potential sources were not evaluated. Sandu et al. [41] focused on the REEs and some trace element geology and geochemistry evaluation in river sediments in the Bistricioara hydrographic basin (Eastern Carpathian Mountains, Romania). In this case, the investigated remote location is less likely to be influenced by anthropogenic urban contributions.

The present study aimed both to evaluate the distribution of potentially toxic heavy metals and rare earth elements and to assess the anthropogenic impact on lake sediments from Podu Iloaiei Dam Lake. The investigated lake is located on the Bahluet River from the Bahlui river basin and is considered as one of the most anthropized hydrological basins of the country in north-eastern Romania. The evaluation of possible contamination of the lake water and sediment is of the utmost importance since it is an important water resource for fish farming in the region. The objectives of this study were: (1) to investigate the spatial distribution characteristics of 15 trace elements (TEs), 8 refractory elements (REs), and 15 rare earth elements (REEs) in Podu Iloaiei Dam Lake sediments in north-eastern Romania; (2) to evaluate element sources in sediments and driving environmental processes using multivariate statistical methods; (3) to assess the pollution and to identify the harmful potential of these elements in the investigated area. The results obtained in this study may provide a reliable scientific database for the estimation of the local ecological status and implementation of pollution remediation strategies.

## 2. Materials and Methods

### 2.1. Site Description and Sample Collection

**Figure 1:**
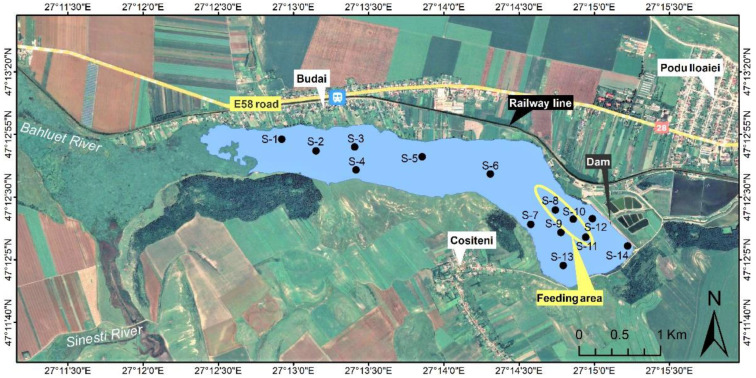
Map of the Podu Iloaiei Dam Lake and the distribution of sediment sampling sites (the Image © 2022 CNES/Airbus from Google Earth Pro was incorporated in the present figure).

The Podu Iloaiei Dam Lake is located on the Bahluet River from the Bahlui hydrographic basin, north-eastern Romania. More geographical details of the hydrographic area are elsewhere reported [42]. The surroundings of the lake and the sampling points are presented in Figure 1. In general, the water level of Podu Iloaiei Dam Lake (water surface of 251 ha) fluctuates considerably throughout the year, with lower values during the summer (June, July, and August) and higher values during major rainfall events in the autumn (September, October, and November), with an average annual rainfall of 589 mm. In 2017 (the year of sampling), the highest average precipitation values were recorded in May (44 mm) and June (54 mm). The lowest level of precipitation was recorded in October, with an average of 27 mm. The Podu Iloaiei Dam Lake is mainly used for fish farming and is also an important source of water for fish ponds downstream of the dam [43]. The water is drained for the wintertime.

In the Prut–Barlad hydrographic area, including the Bahlui hydrographic basin, agricultural (71%), forest (14%), and residential (12%) areas are the dominant forms of land use. According to the estimate of the National Institute of Statistics, only a small area is occupied by surface waters (3%) [44].

A total number of 28 sediment samples were collected from Podu Iloaiei Dam Lake within s two session measurement, on 16 July 2017 and 5 October 2017, and the distribution of the sediment sampling sites is presented in Figure 1. Data on 88 water samples collected during the same sampling sessions are presented elsewhere [42].

### 2.2. Sample Preparation of the Sediment

The solid-state dissolution procedure and inductive coupled plasma-mass spectrometry (ICP-MS) analyses were applied for the metal elemental analysis in the sediment samples. Sodium peroxide (Na_2_O_2_) was used as the oxidizing agent, as it is one of the strongest available [45,46]. A ratio of 1 to 6.5 for sample to Na_2_O_2_ reagent proved to be suitable for complete sample dissolution.

The sediment samples were dried slowly and, subsequently, were finely grinded using an agate mortar and pestle. Around 0.1 g of sediment sample was weighed, and 50 µL of internal standard solution (Indium—200 µg g^−1^) was added to each sample. The samples were further mixed with the oxidizing agent, followed by transfer to the porcelain crucibles lined with aluminum foil. Thermal processing was performed at 460 °C. After cooling, the sintering mix was dissolved in HNO_3_ 3% solution prepared in deionized water and stored in closed polypropylene vials until analysis.

### 2.3. Chemical Analysis

The concentrations of trace elements (TEs: Li, Be, Mn, Co, Zn, Ga, As, Rb, Zr, Cd, Ba, Tl, Pb, Bi, and U), refractory elements (REs: V, Cr, Nb, Mo, Hf, Ta, W, and Ir), and rare earth elements (REEs, divided in Light REEs—LREEs: La, Ce, Pr, Nd, Sm, and Eu; and Heavy REEs—HREEs: Y, Gd, Tb, Dy, Ho, Er, Tm, Yb, and Lu) in the sediment samples were determined by the ICP-MS technique using the ICP-MS 7700X system (Agilent Technologies, Santa Clara, CA, USA).

Analyte quantification was performed by an internal standard calibration procedure. Certipur^®^ Indium ICP standard from Merck KGaA (Darmstadt, Germany) was used as an internal standard. Certipur^®^ ICP multi-element standard solution XXI for MS (Merck KGaA, Darmstadt, Germany) was used for TE quantification, Specpure^®^ refractory metals plasma standard solution (Alfa Aesar—Thermo Fisher GmbH, Erlenbachweg, Kandel, Germany) was used for RE quantification, and TraceCERT^®^ rare earth element mix for ICP (Sigma-Aldrich—Merck KGaA, Darmstadt, Germany) was used for REE quantification. Nitric acid 65% Suprapur^®^ (Merck KGaA, Darmstadt, Germany) was used for HNO_3_ 3% preparation. Ultrapure water obtained from an Advantage A10 water purification system (Milli-Q—Merck-Millipore, Darmstadt, Germany) was used for standards and sample dilution.

Special emphasis was placed on carefully controlling the entire array of preparatory and analysis steps in order to prevent the possible contributions brought by sample loss or contamination. For quality control assurance, blanks and control samples were frequently analyzed.

### 2.4. Statistical Analysis

#### 2.4.1. Statistical Approaches for Source Assessment

The geostatistical approach was used in the present work to comprehensively describe the variation of elements concentration in the sediment of Podu Iloaiei Dam Lake. For the quantified elements, analysis of the geospatial distribution was used in order to discriminate between potential contributions of natural or anthropogenic sources. Google Earth Pro and ArcMap (included in the ArcGIS 10.2 package) programs were used for geospatial distribution analyses. For data processing inverse distance weighting (IDW), interpolation was used.

Pearson correlation analysis and principal component analysis (PCA) were used to identify the possible sources of elements in sediment samples from Podu Iloaiei Dam Lake. Varimax normalized PCA was used to determine the association of the different variables by reducing the dimensionality of the dataset. The concentrations of the quantified elements from sediment samples were used as variables for the PCA. The validity of the PCA was checked with the Kaiser-Meyer-Olkin (KMO) statistic and Bartlett’s test using SPSS (version 22.0). The PCA was performed to determine possible chemical associations that could explain TE, RE, and REE behaviors and the relationships between them.

The Kolmogorov-Smirnov test, carried out in OriginLabPro 2022, was performed to check for possible differences between the sampling sessions. Differences were considered of significance at the level of *p* < 0.05.

The correlation matrix, including Pearson correlation coefficient values, pie charts, pollution indices heat map representations, and the boxplots of the potentially harmful elements, were graphically represented in OriginLabPro 2022.

#### 2.4.2. REEs Normalization and Anomalies

The normalization to Chondrite [17] and the upper continental crust (UCC) [47] was performed for the evaluation of the REE distribution in the investigated sediment samples and for a better comparison of the two sampling sessions. Although North American Shell Composites (NASC), Post-Archean Australian Shale (PAAS), and European Shell (ES) normalizations were also performed, in terms of distributions, the differences compared to UCC were almost insignificant and therefore were of no interest in the present study.

The Ce and Gd anomalies were estimated as identified in the literature with Equation (1) for Ce/Ce* [12,21,48,49] and Equation (2) for Gd/Gd* [22,48,50]:

(1) $$\mathrm{Ce}/{\mathrm{Ce}}^{*}\;=\;2\;{\mathrm{Ce}}_{N}/\left(La_{N}+Pr_{N}\right)$$

(2) $$\mathrm{Gd}/{\mathrm{Gd}}^{*}\;=\;3\;{\mathrm{Gd}}_{N}/\left(Sm_{N}+2\;Tb_{N}\right)$$

where *N* represents the normalized concentrations to the UCC values [47].

The bell-shape index (*BSI*) was evaluated using the formula presented in Equation (3) [12], with the main aim to determine the enrichment of the environment in medium *REEs*. The *lREE*, *mREE*, and *hREE* factors were determined using Equations (4)–(6), respectively. The concentration of *REEs* was normalized to UCC, and Ce was excluded from the *lREE* factor calculation due to anomalous behavior.

(3) BSI = 2 mREEs/(lREEs+hREEs)

(4) $$lREEs\;=\;(La_{N}+Pr_{N}+Nd_{N})/3$$

(5) $$mREEs\;=\;(Sm_{N}+Eu_{N}+Gd_{N}+Tb_{N}+Dy_{N})/5$$

(6) $$hREEs\;=\;(Ho_{N}+Er_{N}+Tm_{N}+Yb_{N}+Lu_{N})/5$$

#### 2.4.3. Sediment Contamination Assessment

The contamination factor (*CF*), pollution load index (PLI), geoaccumulation index (*I_geo_*), and enrichment factor (*EF*) were estimated in the present work in order to assess the Podu Iloaiei Dam Lake sediment contamination with TEs and REs. The equation and the criteria for assessing the pollution indices are presented in Table 1. In the estimation of the *EF*, Mn was used as a reference element to determine the anthropogenic element enrichment.

Because background element concentrations in the Podu Iloaiei Dam Lake sediment are not available, the Average Shale Values (ASVs) from [51] were used in the present work to calculate the pollution indices.

The potential of the contaminants to cause adverse effects on aquatic organisms was determined by comparing the concentrations of Cr, Zn, As, Pb, and Cd with the Sediment Quality Guideline (SQG) values for freshwater sediments.

## 3. Results and Discussion

### 3.1. Elements Concentration in Sediment Samples

Table 2 shows the mean, minimum, and maximum concentration (μg g^−1^) values for the TEs, REs, and REEs determined in the sediment samples collected in July and October 2017 from Podu Iloaiei Dam Lake. REE concentration values in the water and the water-soluble fraction from sediments (all below the limits of quantification) are presented in Table S1 in the Supplementary Materials (SM).

In the sediment samples, the average of ∑TE concentrations was higher in July (1755 µg g^−1^) compared to October (1540 µg g^−1^). For the ∑RE concentrations, the difference was almost insignificant, with 276 µg g^−1^ in July versus 262 µg g^−1^ in October. While variation of the emission sources between the sampling sessions could be responsible for TE abundances, more stable sources could explain the RE behavior. For the ∑REEs, an opposite trend could be observed with a higher average concentration in October. However, more detailed aspects regarding the determined concentrations of REEs and potential sources will be discussed in Section 3.3.

Regarding the TE concentrations in both sampling sessions, the highest levels were found for Mn, followed by Ba, Zn, and Rb, an observation which may indicate a natural accumulation process [55]. Zinc concentration average values (132 µg g^−1^ in July and 124 µg g^−1^ in October) were consistent with the values reported by Sandu et al. [41] for stream sediments (Bistricioara, Romania). The mean concentration of Cr was 103 µg g^−1^ in July and 94.6 µg g^−1^ in October (Table 2), which are higher than the values reported in lake sediments from Bafa Lake, Turkey (75.90 ± 13.22 µg g^−1^ in summer season and 80.97 ± 29.74 µg g^−1^ in autumn season) [3].

The present study reports significantly higher concentrations for Cd (0.73–1.98 μg g^−1^ for July and 1.35–2.06 μg g^−1^ for October) compared with sediment from Lake Balaton, Hungary (0.1–0.7 μg g^−1^) reported by Nguyen et al. [56]. In the Nguyen et al. [56] study, lower concentrations for Cr (5.7–66 μg g^−1^) and Mn (160–760 μg g^−1^), similar concentrations for Co (1.7–17 μg g^−1^) and Zn (13–150 μg g^−1^), and higher concentrations for Pb (2.4–160 μg g^−1^) were presented. By comparison with a remote location, Bory Tucholskie National Park, Poland [49], used for the geochemical background values estimation, the REE concentrations in Podu Iloaiei Dam Lake sediment were higher and may indicate anthropogenic contribution in our case.

The Kolmogorov-Smirnov test indicated different distributions for Bi, Ce, Hf, Ir, Ta, U, Zr, Ba, Be, and Mn between the two sampling sessions. The element concentration range was considerably wider in July compared to October, and this might suggest a mixed source contribution to the element content in July (Table 2).

**Figure 2:**
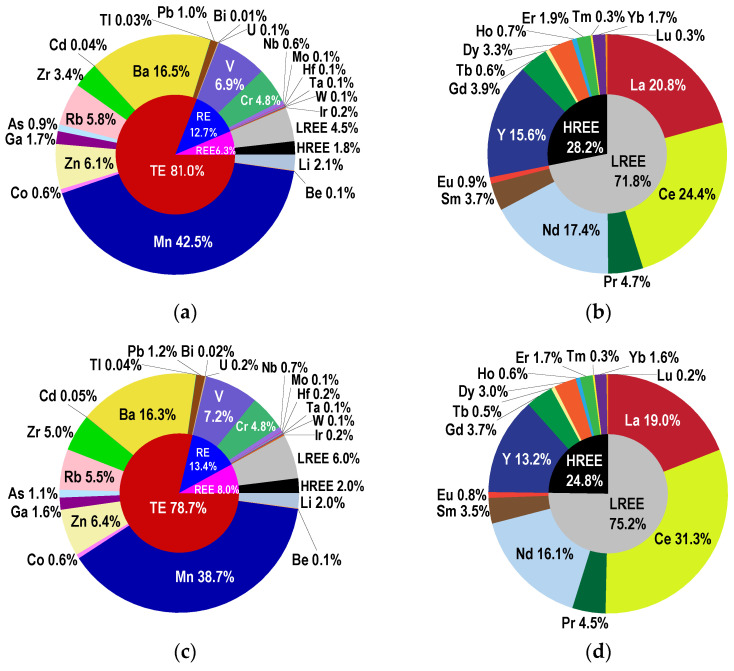
Relative contribution averages for July ((**a**)—total quantified elements, (**b**)—rare earth elements) and October ((**c**)—total quantified elements, (**d**)—rare earth elements) in sediment samples collected from Podu Iloaiei Dam Lake.

The relative distributions of quantified elements in July and October are presented in Figure 2. TEs, REs, and REEs contributed with 81.0%, 12.7%, and 6.3% in July (Figure 2a) and with 78.7%, 13.4%, and 8.0% in October (Figure 2c). In the ΣREEs, the LREEs and HREEs contributed with 71.8% and 28.2% in July (Figure 2b), respectively, 75.2% and 24.8% in October (Figure 2d). A decrease in TE contribution was observed from July to October, together with a slight increase for REEs. This is related to the increase in Ce contribution to the REEs from 24.4% in July to 31.3% in October. The individual contribution of other elements to ΣREEs was relatively constant.

The relative contributions of TEs for both sampling sessions varied in the following order: Mn > Ba > Zn > Rb > Zr > Li > Ga > Pb > As > Co > U > Be > Cd > Tl > Bi. As presented in Figure 2a,c, for Mn contribution, a slight decrease was observed from July to October.

For the RE group, the relative contributions varied in the following order: V > Cr > Nb > Ir > Hf > Mo > W > Ta, with V presenting a relatively constant contribution. For REEs, particularly Ce, La and Nd brought high contributions to the LREEs, while Y represented about half of the HREEs in both sampling sessions.

### 3.2. Spatial Distribution of Elements

**Figure 3:**
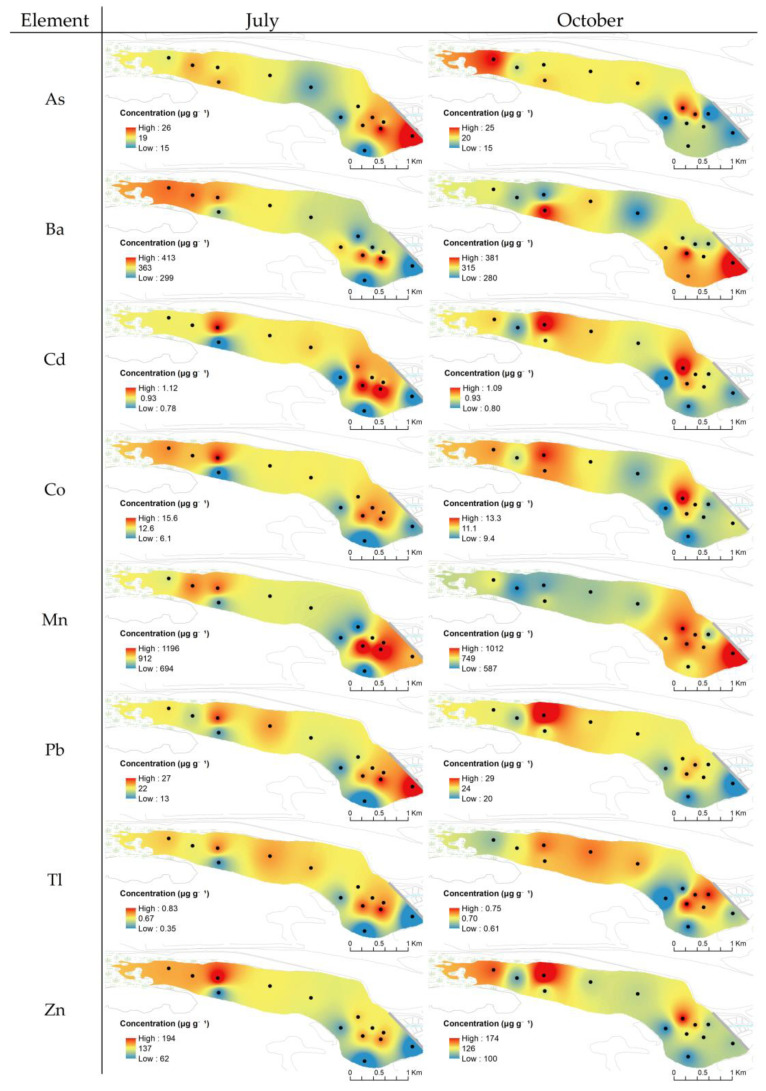

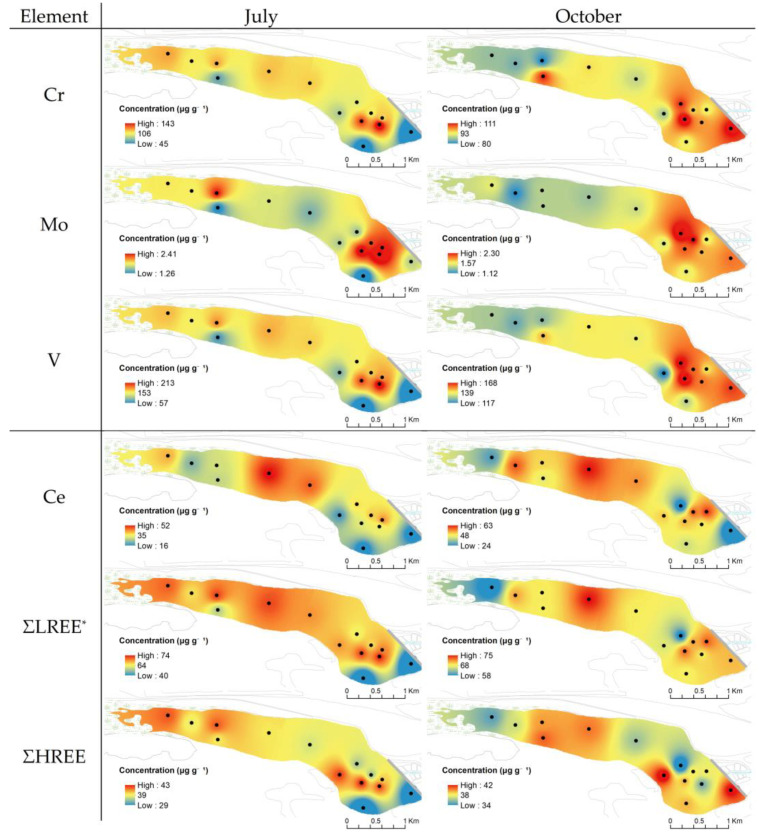
Spatial distribution of selected TEs (As, Ba, Cd, Co, Mn, Pb, Tl, and Zn), REs (Cr, Mo, and V) and Ce, ΣLREE*, and ΣHREE in the sediment of Podu Iloaiei Dam Lake for the July and October sampling sessions.

IDW interpolation was performed on the element concentrations as a mapping method to obtain information about their spatial distributions. The selected TEs (Mn, Co, Zn, As, Cd, Ba, Tl, and Pb), REs (V, Cr, and Mo), Ce, LREEs* (LREEs without Ce), and HREEs spatial distributions in the sediment of Podu Iloaiei Dam Lake for July and October are presented in Figure 3. The distributions of other TEs (Li, Be, Ga, Rb, Zr, Bi, and U) and REs (Nb, Hf, Ta, W, and Ir) are presented in Figure S1 from the SM.

As observed from Figure 3, for Co, Zn, Cd, Tl, Pb, V, Cr, and Mo, lower concentration levels were identified in the south of the lake (S-4, S-7, and S-13 in Figure 1), close to the meadow and forest. Higher concentrations of these elements are observed in the middle and the north lake areas (S-3, S-5, and S-6 in Figure 1), with more susceptibility to be influenced by contributions of the residential area, railway, and transport road.

The profile of the spatial distribution of Mn, Ba, V, Cr, Mo, HREEs, and LREEs may indicate a displacement of the sediment from the input water area (in the west side) especially in July.

Similar patterns were observed in July for the distributions of As, Cd, and Pb. In October, As seemed to have a more pronounced contribution from the river water source, while Cd and Pb seemed to be associated with contributions from the road and railway traffic. Cadmium, Pb, and other elements’ distribution in soil samples collected from nearby roads were also highlighted in recent studies [57,58]. High concentrations of these elements were observed in the feeding area and near the dam. This may indicate the existence of a punctual and persistent source of pollution related to food supplied for fish in both sampling sessions.

Wastewater discharge and traffic might be important contributors for Co and Zn in both sampling sessions. Additionally, the feeding area presented higher concentrations of these elements.

Moreover, it is worth mentioning that V, Cr, and Mo presented higher concentrations in the feeding zone in both sampling sessions, with the higher concentration area being broader in October compared to July.

A complementary spatial distribution of Mn and Ce was observed. This indicated that the oxidative dissolution of Mn from the sediment particles can generate Ce enrichment from Ce^4+^ [59,60]. The cerium concentration showed the highest variability along the lake and between the two sampling periods, indicating different transport and deposition processes of the sediments in addition to redox conditions of the lake.

Element concentration variability was higher in July compared to October, with the coefficients of variation from 10 to 33% in July (19% in average) and from 5 to 26% in October (10% in average). This variability indicated that the urbanization, accumulation, and distribution of the potential toxic elements in lake sediments may lead to an increased spatial heterogeneity. For almost all REEs, the coefficient of variation values were higher in July (10 to 18%) compared to October (5 to 8%). These aspects indicated a homogeneous spatial distribution for these elements, except for Ce (with 33% in July and 26% in October). Differences in the input water quality, the weathering and leaching from soil surface, the local biochemical processes in the area with low water levels, and direct anthropogenic pressure could be important factors controlling the variability in the chemical composition of sediment samples from July. In October, the increase of the minimum concentration led to a reduced variability.

For measured REEs, the variations observed between sampling sessions may be related to the input water source, which may change the pH and salinity of the lake, seasonal changes in water redox conditions, and also due to an enhancement of the adsorption–desorption reactions and REEs complexation processes [15].

### 3.3. REEs Normalization and Anomalies

**Figure 4:**
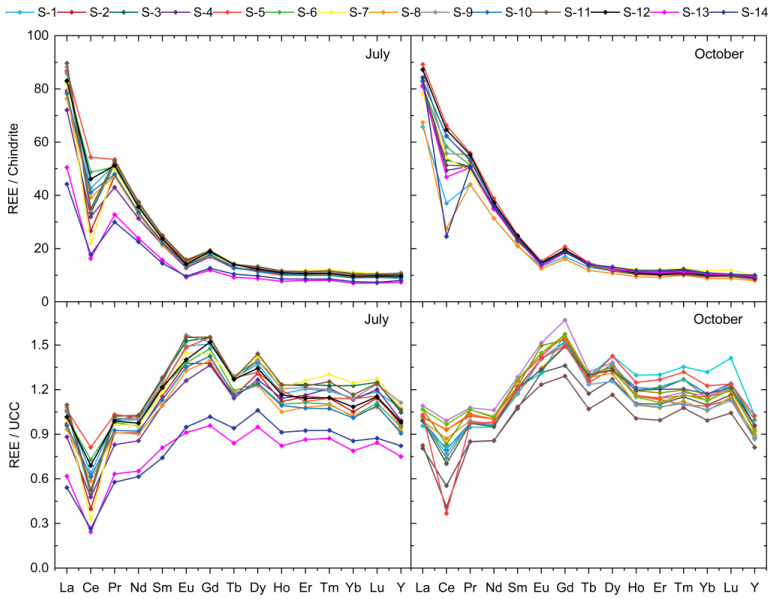
The Chondrite-(**up**) and UCC-normalized (**down**) REE concentration distribution in the investigated samples for both sampling sessions.

The distribution of Chondrite- and UCC-normalized REE concentrations for all sediment samples is presented in Figure 4. Negative anomalies can be observed for Ce and Eu elements, while the positive anomaly is identified for Gd in the Chondrite-normalized distribution. The UCC-normalized REE concentrations show non-flat patters, indicating that some fractionation may be present. The similar profiles of the REE normalized concentrations over the investigated area indicated common sources in sediments [16]. However, the presence of the Eu negative anomaly is relatively common in the upper crust [49,50]. The numerical evaluation of the Ce anomaly was performed using, in Equation (1), the UCC-normalized concentrations of its neighbors, La and Pr. The approach could not be applied for Gd because its left neighbor, Eu, is itself susceptible to anomalous behavior. Therefore, for the Gd anomaly evaluation, the Sm normalized concentration was used in Equation (2).

The negative Ce anomaly (Ce/Ce* < 1) was determined for all investigated samples, with values ranging from 0.33 to 0.78 in July and from 0.37 to 0.92 in October. The individual values of the Ce anomaly are included in Table S2 from the SM. Data presented in Figure 4 show that few samples from the October sampling session (i.e., S-2, S-5, S-10, and S-12 in Figure 1) have an almost insignificant Ce anomaly. It is worthy to mention that S-10 and S-12 samples are associated with nutrient rich feeding areas with higher susceptibility to present more intense bacterial activity (that enhance Ce^3+^ oxidation to Ce^4+^) on the surface of the water in October than in July. Under these circumstances, the sedimentation of Ce^4+^ due to scavenging by Fe-Mn oxide particles [15] may induce a reduced anomaly in the sediment samples, as observed in the present study. No correlation between Ce/Ce* and Mn content was observed for the July samples (Pearson correlation coefficient of −0.0005), while in October the correlation coefficient was as high as −0.7577, reflecting an actually significant negative correlation of the Ce anomaly and the Mn content. The correlation between the Ce anomaly and Mn content is in agreement with other reports [9,12,20,61,62].

Considering that the Mn concentration in sediment samples was fairly similar for both sampling sessions, it is assumed that, in October, the variation of the Ce anomaly was mainly due to the more pronounced oxidation potential of the water body. Only then, the formation of Ce^4+^ is possible, followed by Mn-containing particle scavenging, which finally leads to a diminished Ce anomaly in the sediment due to sedimentation.

A positive Gd anomaly was obtained for both July and October sediment samples. The values obtained for the Gd anomaly varied from 1.15 to 1.24 in July and from 1.18 to 1.28 in October. The individual values are presented in Table S2 from the SM. Although a slight increase from July to October can be observed, the geochemistry and redox condition of the lake were not controlling factors of Gd anomaly. Gadolinium-chelating agents are known to have applications in MRI [11,22,23]. For the investigated dam lake water, micropollution with Gd was associated with the largest medical hub in north-eastern Romania (Iasi, 30 km away from the investigated area, in average with about 10 MRI/1000 inhabitants especially during the last years). Similar results for water resources situated even at about 50 km away from medical hubs or highly populated areas are presented also in other studies [22,48]. Since the use of MRI in medical imaging is continuously growing (see Figure S2 from Supplementary Materials), the Gd micropollution in water resources situated nearby is expected to increase. Additionally, the discharge of treated wastewater into the environment can play a significant role for the positive Gd anomaly, as Gd-chelating agents are not successfully being removed in water treatment plants [22]. Moreover, Gd can have potential toxic effects due to its similar ionic radius, similar electronegativity, and bigger binding affinity to that of Ca^2+^ [15]. It is already suggested that Gd can block the Ca ion channels in cells [48,63] or may alter the catalytic effect of Ca^2+^ in enzymes [63,64].

As presented in Figure 4, for the UCC-normalized REE concentration distribution, medium mass REE concentrations higher than the values of the UCC may indicate an enrichment process. The Bell-Shaped index (*BSI*) was used to numerically evaluate the element enrichment in the investigated dam lake sediment. The individual values of the *BSI* are presented in Table S2, from the Supplementary Materials, together with the values of the Ce and Gd anomalies. The *BSI* values ranging from 1.21 to 1.28 in July (average of 1.25) and from 1.19 to 1.28 in October (average of 1.25) suggested stability in terms of the sampling points and sampling sessions. The enrichment of *m*REEs (Sm, Eu, Gd, Tb, and Dy) associated with phosphate minerals [65] was most probably related to geological processes [16,66,67] but may also indicate anthropogenic sources, such as phosphogypsum pollution [23] or the use of P-fertilizers. Although the anthropogenic contribution cannot be accurately estimated, since about 71% of the surrounding area of the Podu Iloaiei Dam Lake is used for agriculture [44], P-fertilizers can be suggested as a possible important contamination source.

### 3.4. Correlation Analysis and Principal Component Analysis

**Figure 5:**
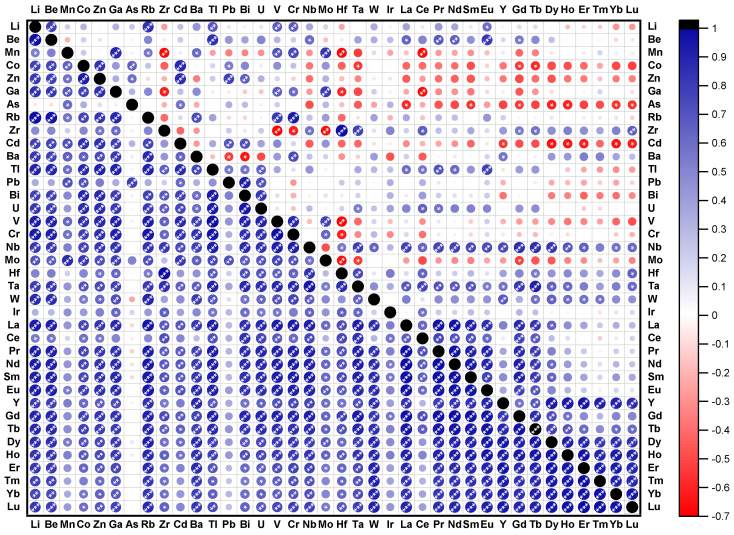
Pearson correlation coefficient matrix for the relationships between the element concentrations in the Podu Iloaiei Dam Lake sediment in July (left) and October (right). The color and the diameter of the circles are related to the correlation coefficient value (according to the color scale on the right) and the *p*-value (inversely proportional), respectively. The number of white stars is related to the *p*-value: * (*p* < 0.05), ** (*p* < 0.01), and *** (*p* < 0.001).

The correlations between the investigated elements (Pearson correlation coefficients) are presented in Figure 5, with major differences observed between the sampling sessions. Significant correlations (*p* < 0.05) were observed for As with Mn (0.60) and Pb (0.79) in July. In October, the correlations extended to Co (0.66), Zn (0.59), and some REEs (La:−0.58, Sm:−0.56, Gd:−0.54, Dy:−0.60, Ho:−0.61, Yb:−0.56, and Lu:−0.57). Cadmium was significantly correlated (p < 0.05) with Co (r = 0.86, 0.89), Zn (r = 0.86, 0.87), Bi (r = 0.79, 0.73), and Pb (r = 0.61, 0.71) in both sampling sessions (Figure 5). Significant correlations at *p* < 0.05 were observed between Mn and Ga (r = 0.69, 0.86), V (r = 0.63, 0.72), Cr (r = 0.76, 0.59), and Mo (r = 0.87, 0.83). Negative significant correlations were observed between Mn and Zr (r = −0.68), Hf (r = −0.68), and Ce (r = −0.69) in October. These observations suggested that the reductive dissolution of Mn from the sediment can generate Ce, Hf, and Zr enrichment because these elements are redox condition sensitive [59,60].

Significant strong correlations at *p* < 0.01 were observed between Zr and Hf, with a correlation coefficient of 0.99 in both sampling sessions. Such behavior is expected since Zr and Hf elements are very similar in their size and chemical properties due to the lanthanide contraction [68].

High values of Pearson coefficients were obtained between REEs (r > 0.81, *p* < 0.05), except for Ce in July. For October, a different behavior was observed, with a clear separation between LREEs and HREEs. Figure 5 shows significant correlations between LREEs (r > 0.77, *p* < 0.05), except for Ce, and between HREEs (r > 0.81, *p* < 0.05), except for Gd and Tb (Figure 5). These last two elements are more likely correlated with LREEs (r > 0.68, *p* < 0.05). In both sampling sessions, Nb and Ta elements were highly correlated with most of the REEs.

**Figure 6:**
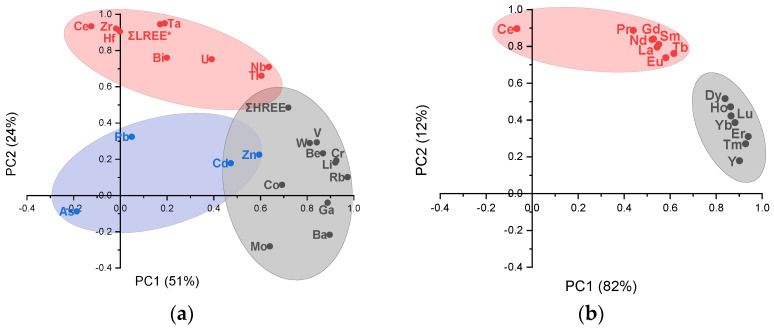
Loading of PC1 versus PC2 factors from TE, RE, Ce, ΣLREE*, and ΣHREE (**a**) and REE (**b**) quantified in the sediment samples of Podu Iloaiei Dam Lake (loading values are presented in Tables S3 and S4 in the Supplementary Materials).

Furthermore, the PCA was performed on the database in order to identify the most important sources for TEs, REs, and REEs in the sediment of Podu Iloaiei Dam Lake. The results from the PCA applied to the database are presented in Figure 6 and Tables S3 and S4 from the Supplementary Materials. For optimal identification of the statistical solutions, the PCA was performed on the data set obtained by merging the results from both sampling sessions. The elements were divided into two groups, the first one including TEs, REs, Ce, LREEs*, and HREEs, while the second group was represented by all REEs. The measure of sampling adequacy by KMO statistics provided the values of 0.62 for the first group and 0.85 for the last one, while the significance of Bartlett’s test of sphericity was < 0.001 in both cases. Both tests led to the conclusion that the database size was suitable to be evaluated by the PCA. The number of factors with eigenvalues greater than 1.0 changed from three for the first group of elements to two for the second one, with 85.6% and, respectively, 93.5% of the variance explained. As presented in Table 3, the factor loadings were classified into strong and moderate, with the loading values of >0.75 and 0.75–0.50, respectively.

Figure 6 presents the loading of PC1 versus PC2 factors from the PCA applied to the investigated lake. Factor 1 in the first group of elements (TEs, REs, Ce, LREEs*, HREEs) had strong loadings of Rb, Cr, Li, Ba, Ga, Be, V, and W and moderate loadings of HREEs, Co, Mn, Mo, Nb, Tl, and Zn. This factor can be attributed to a mixed source of rock weathering and leaching from the soil surface. Work performed in the research group allowed for suggesting a potential additional contribution to this factor from atmospheric deposition (dry or wet). Stormwater residuals from the high traffic road located in the proximity of the lake can be another source [69,70]. In Factor 1, strong loading was observed for W (0.81) with moderate loadings for Mo (0.64) and Zn (0.59). This agrees with other studies that indicated W, Mo, and Zn to be tracers of anthropogenic pollution from the urban road dust [69,70].

Factor 2 was comprised of Ta, LREEs*, Ce, Hf, Zr, Bi, U, Nb, and Tl, corresponding to pedological characteristics of the investigated area. This may also highlight the Ce, Hf, and Zr elements sensitivity to the redox status of the sediment [71]. Factor 3 had strong loadings of Pb, As, and Cd with moderate loadings of Zn, Co, and Mo. This factor can be attributed to mixed sources of wastewater discharge, leaching from agricultural lands, or waste accumulation in unauthorized areas surrounding the Bahluet River.

The PCA analysis applied to the REE database discriminated two factors. Factor 1 had strong loadings of HREEs (Er, Tm, Y, Yb, Lu, Ho, and Dy), except for Gd and Tb, which presented strong loadings in Factor 2 among the LREEs (Ce, Pr, Nd, Sm, and La). A slight distancing of Ce from the Factor 2 elements can be observed in Figure 6b, indicating additional specific influences.

### 3.5. Contamination Assessment and Potentially Harmful Elements

**Figure 7:**
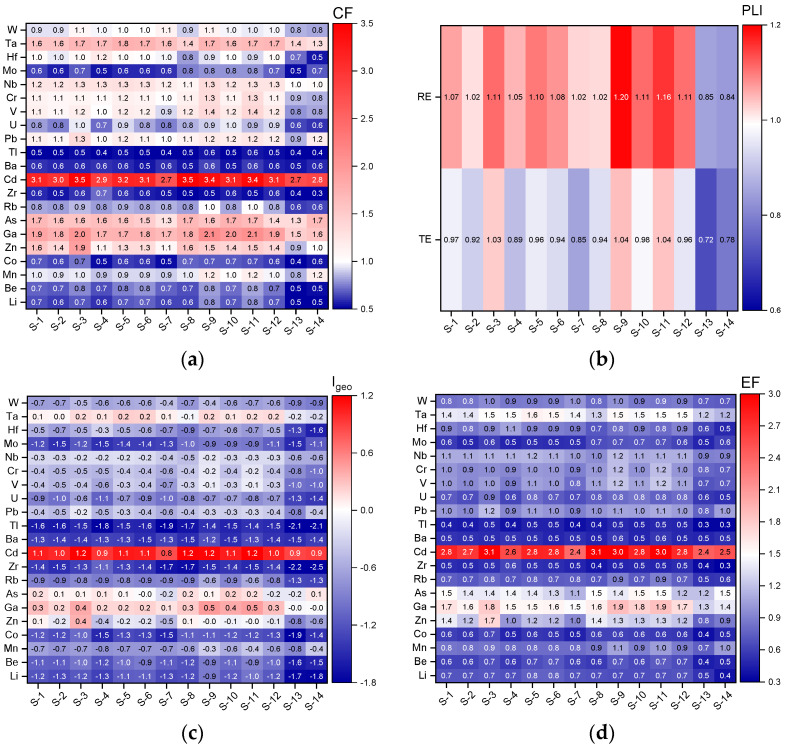
Contamination factors (*CF*s), (**a**) pollution load indices (PLIs), (**b**) geoaccumulation indices (*I_geo_*s) (**c**), and enrichment factors (*EF*s) (**d**) calculated for the sediment sampling points from Podu Iloaiei Lake.

Figure 7a presents the contamination degree assessment of the individual contaminant, by CF, at the interest sampling points. For a high number of elements (Li, Be, Co, Rb, Zr, Ba, Tl, U, and Mo) the contamination factor values were smaller than 1, indicating a low degree of contamination in the sediment samples. Vanadium, Cr, Nb, and Hf showed moderate contamination, with CF values close to 1. Low or moderate degrees of contamination (CF < 3) were observed for all elements, except for Cd, in all sampling points. CFs for Cd showed considerable contamination (CF = 3 ÷ 6) at almost all the sampling points. The S-4, S-7, S-13, and S-14 sampling points were exceptions, with CF values between considerable and moderate contamination (CF = 1 ÷ 3). The variation in CFs of Cd, a very toxic element in the aquatic ecosystem, which tends to bioaccumulate in sediment [72], reflected different source contributions between the sampling points. Cadmium contamination in Podu Iloaiei Dam Lake may be caused by fuel used for fishing boats or phosphate fertilizers and pesticides used in agricultural areas [3]. The railway line (606 magistral) and high road traffic (E58 road), located near the lake, might be important sources too.

The *CF*s of Zn, Ga, As, and Pb showed moderate contamination for all sampling points, with the highest values obtained for S-8, S-9, S-10, S-11 and S-12, corresponding to the feeding area. Natural weathering of minerals is also a known source for Zn [73]. The use of Pb-As insecticides and arsenic-based herbicides [74] on agricultural land in neighboring areas could contribute to the Pb and As concentrations in the sediment from Podu Iloaiei Dam Lake.

Figure 7b presents the results from the PLI analysis, with values ranging from 0.72 to 1.04 for TEs, and from 0.82 to 1.20 for REs. Several sampling points (S-1, S-9, and S-11 in Figure 1) from the Podu Iloaiei Dam Lake had PLI values for TEs very close to 1, indicating a slightly progressive deterioration of the environment in these locations. With the exception of the S-13 and S-14 samples, the PLI values for REs were above 1 in all locations, mainly due to sediment contamination with V, Cr (from fuel combustion, [75,76]), Nb, and Ta (from e-wastes, [77]). The sampling points associated with the feeding area (S-09, S-10, S-11, and S-12) seemed to have some of the highest PLI values for REs, highlighting a supplementary pollution source. This may be related to boat fuel, which is used more frequently in the feeding area. Domestic wastewater from settlements with no sewage infrastructure near S-1, S-3, and S-5 could be the main source of pollutants in this part of the lake.

The PLI values obtained for the S-1 and S-2 sampling points (Figure 1) located near a large area covered by plants highlighted the potential role of aquatic plants in absorbing and accumulating heavy metals [3].

The results obtained with the geoaccumulation index (*I_geo_*) are shown in Figure 7c. The *I_geo_* values indicate that the sediment was uncontaminated (*I_geo_* ≤ 0) for most of the investigated TEs and Res. None to moderate contamination (*I_geo_* = 0 ÷ 1) with Zn, Ga, As, and Ta was observed. For Cd, element moderate contamination (*I_geo_* = 1 ÷ 2) was found at 10 sampling locations, confirming the contributions from anthropogenic pollution sources. Only at the S-4, S-7, S-13, and S-14 sampling locations, the *I_geo_* values for Cd were below 1. The highest *I_geo_* values for Zn and Ga were found at S-3. Sediments at the S-9, S-10, and S-11 locations were hinted as uncontaminated to moderately contaminated with Ga, As, and Ta. In specific agricultural activities related to crops of sunflower, wheat, corn, and rapeseed, fruit-bearing orchards in the area, the use of agrochemicals (e.g., herbicides, insecticides) generally used at the European level can be responsible for As pollution, as suggested by Defarge et al. [74]. Another possible source for As in north-eastern Romania may be represented by atmospheric depositions. Galon-Negru et al. [78] reported the largest concentration of As in summer and autumn, associated with a bursting increase in the coal combustion process. Moreover, the study showed that the water-soluble As from the fine particulate matter in the Iasi area had the largest contribution to inhalation and carcinogenicity risks. Possible contamination with Ga and Ta may be attributed to improper management of electronics in the residential area, with these elements being assigned as contaminants from e-wastes [79].

The influence of anthropogenic sources on the elements in the sediment samples was also assessed by determining the *EF* values. The calculated element EFs are shown in Figure 7d. For all sampling points, *EF* values less than 1.5 were obtained for the investigated elements, indicating their most probable natural origin, with few exceptions being observed for Zn, Ga, As, and Ta. Cadmium showed the highest *EF* values, with moderate anthropogenic modification (3 < *EF* < 5) for the S-3, S-8, S-9, and S-11 sampling points and minor anthropogenic modification (1.5 < *EF* < 3) for the other ten sampling points from Podu Iloaiei Dam Lake. The obtained results confirmed the anthropogenic source of Cd, with emissions associated mainly with fuel used for boats, railways, and road traffic.

**Figure 8:**
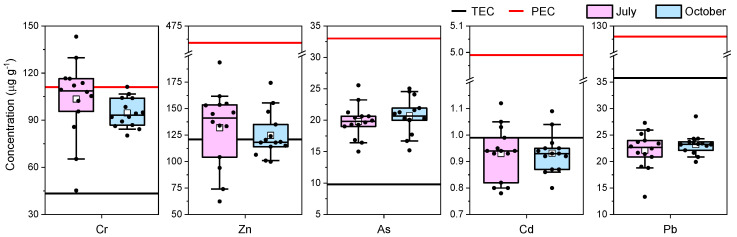
Box plots of the potentially harmful element (Cr, Zn, As, Cd, and Pb) concentration in the Podu Iloaiei Lake sediment, along with the threshold effect concentration (TEC) and probable effect concentration (PEC)) (thin black line—median; white square—mean; box—25–75% percentiles; length of the whiskers plot—10% and 90% of observed concentrations).

The potentially harmful element (Cr, Zn, As, Cd, and Pb) concentration in the sediment samples of Podu Iloaiei Dam Lake, along with threshold effect concentration (TEC) and probable effect concentration (PEC) for each element, are shown in Figure 8.

Among the investigated elements, Cd and Pb showed concentrations that were mainly below TEC in all samples. The obtained results indicated that these elements are very unlikely to pose a threat to the organisms living in the bottom of the aquatic system [3] in the investigated location. For Cr and As, the concentrations were higher than the TECs for all sampling points, both in the July and October sampling sessions. The occurrence of different anthropogenic sources or processes during the two sampling sessions could be responsible for the variability in Zn concentrations, which were higher than the TEC.

The concentrations of Zn, As, Cd, and Pb did not exceed the PEC values for any of the investigated samples. Values higher than the PEC thresholds were identified for Cr in several samples (five sampling points in July and one in October), and such behavior may hint that Cr is one of the most important elements that could lead to toxicity in the investigated area. The sources of Cr pollution in Podu Iloaiei Dam Lake may be related to domestic wastewater from villages without centralized sewerage and fuel combustion associated with road traffic, both sources being located in the northern part of the lake.

## 4. Conclusions

High variabilities of TE and RE concentrations in the Podu Iloaiei Dam Lake sediments were identified. For both TEs and REs, indication of their main geogenic origin was obtained. The concentrations of Pb, As, Cd, and Zn were mainly related to anthropogenic sources.

The REEs had low variability, except for Ce. Normalized distributions of REEs and the observation of an enrichment of *m*REEs (Sm, Eu, Gd, Tb, and Dy), evaluated by *BSI*, indicated shared sources such as phosphates that may have both natural and anthropogenic origins. The statistical evaluation of redox-sensitive elements, such as Ce and Mn, and the Ce anomaly highlighted a higher oxidizing potential of the aqueous environment in October compared to July. A positive Gd anomaly was observed for the Chondrite-normalized concentration in both the July and October sampling sessions, which indicated micropollution and may be related to the use of Gd-chelating agents in MRI, a medical investigation technique with a growing use in the Iasi medical hub.

The assessment of the pollution indices emphasized that the sediment samples were highly enriched in Cd and contaminated with elements such as Zn, Ga, As, Pb, V, Cr, Nb, and Ta, related to anthropogenic sources. Sediment quality guidelines indicated that Cr posed a considerable threat to the aquatic biota.

The present study brought high contribution to understanding the specific sources of REEs, TEs, and REs in the aquatic system used for intensive fish farming, from north-eastern Romania. The use of agrochemicals (fertilizers, herbicides, and insecticides), road and railway traffic, and domestic wastewater discharge were found to be the major contributors to the potential harmful element contents in the Podu Iloaiei Dam Lake sediment.

A close monitorization of the Bahluet River, which is the main water input on the investigated Podu Iloaiei Lake, and even extending the investigation on the Bahlui hydrographic basin may be necessary in order to fully understand the evolution of metal pollutants and micropollutants.

## Figures and Tables

**Table 1:** Sediment pollution indices and evaluation criteria for potential contamination assessment.

Pollution Index	Equation	Evaluation Criteria
Contamination factor (*CF*) [34,52,53]	$CF=\frac{C_{n}}{B_{n}}$	<1, low degree of contamination 1 ÷ 3, moderate degree of contamination 3 ÷ 6, considerable degree of contamination >6, very high degree of contamination
Pollution load index (PLI) [32,33]	PLI= ${\left(CF_{1}\times CF_{2}\times \ldots \times CF_{n}\right)}^{\frac{1}{n}}$	0, no pollution 0 ÷ 1, only baseline pollutants >1, progressive deterioration
Geoaccumulation index (*I_geo_*) [1,37,54]	$I_{geo}=log_{2}\frac{C_{n}}{1.5\;\times \;B_{n}}$	≤0, no contamination 0 ÷ 1, none to moderate contamination 1 ÷ 2, moderate contamination 2 ÷ 3, moderate to heavy contamination 3 ÷ 4, heavy contamination 4 ÷ 5, heavy to extreme contamination >5, extreme contamination
Enrichmentfactor (*EF*) [1,2,31,34,36]	$EF=\frac{C_{n}/C_{Mn}}{B_{n}/B_{Mn}}$	0 ÷ 1.5, natural processes 1.5 ÷ 3, minor anthropogenic modification 3 ÷ 5, moderate anthropogenic modification 5 ÷ 10, severe anthropogenic modification >10, very severe anthropogenic modification

Note: *C_n_* = measured concentration of element *n*; *B_n_* = background concentration of element *n*; *C_Mn_* = measured concentration of *Mn*; *B_Mn_* = background concentration of *Mn*.

**Table 2:** Descriptive statistics for the TE, RE, and REE concentrations in sediment (μg g^−1^, n = 14/session) samples collected in July and October 2017 from Podu Iloaiei Dam Lake, Romania. Data are presented as mean (minimum–maximum).

Element	July	October
As	19.8 (15.0–25.5)	20.7 (15.2–25.0)
Ba	358 (299–413)	319 (280–381)
Be	2.25 (1.23–2.97)	1.96 (1.60–2.25)
Bi	0.27 (0.12–0.35)	0.33 (0.25–0.43)
Cd	1.62 (0.73–1.98)	1.81 (1.35–2.06)
Co	12.4 (6.11–15.6)	11.1 (9.40–13.3)
Ga	37.1 (19.8–47.3)	32.0 (25.6–39.6)
Li	45.6 (20.4–62.9)	39.9 (31.2–47.8)
Mn	922 (694–1196)	757 (587–1012)
Pb	22.1 (13.3–27.3)	23.2 (19.9–28.5)
Rb	125 (59.2–171)	107 (94.4–130)
Tl	0.65 (0.35–0.83)	0.70 (0.61–0.75)
U	2.87 (1.58–3.49)	3.20 (2.75–3.68)
Zn	132 (62.3–194)	125 (100–174)
Zr	74.0 (25.5–115)	97.8 (65.3–127)
ΣTE	1755 (1241–2205)	1540 (1311–1830)
Cr	103 (45.3–143)	94.6 (80.2–111)
Hf	2.27 (0.90–3.33)	3.02 (2.05–3.84)
Ir	3.30 (1.68–4.78)	4.38(3.40–6.03)
Mo	1.83 (1.26–2.41)	1.62 (1.12–2.30)
Nb	13.2 (9.15–15.1)	13.5 (11.1–14.7)
Ta	1.19 (0.81–1.38)	1.38 (1.11–1.49)
V	149 (57.3–213)	142 (117–168)
W	1.79 (1.12–2.35)	1.72 (1.39–1.95)
ΣRE	276 (118–383)	262 (231–306)
Ce	33.5 (15.5–52.0)	48.8 (23.5–63.5)
Eu	1.20 (0.80–1.38)	1.23 (1.09–1.33)
La	28.5 (16.2–32.9)	29.6 (24.1–32.7)
Nd	23.8 (16.0–26.7)	25.1 (22.3–27.6)
Pr	6.48 (4.10–7.33)	7.00 (6.03–7.65)
Sm	5.10 (3.34–5.76)	5.41 (4.83–5.78)
ΣLREE	98.5 (57.5–124)	117 (85.3–138)
Dy	4.49 (3.32–5.05)	4.66 (4.08–4.99)
Er	2.59 (1.99–2.90)	2.66 (2.29–2.99)
Gd	5.33 (3.64–5.90)	5.75 (4.91–6.34)
Ho	0.89 (0.66–0.99)	0.93 (0.80–1.04)
Lu	0.36 (0.27–0.41)	0.38 (0.33–0.45)
Tb	0.76 (0.54–0.83)	0.81 (0.68–0.84)
Tm	0.37 (0.29–0.43)	0.39 (0.36–0.45)
Y	21.4 (16.5–24.5)	20.5 (17.8–22.5)
Yb	2.36 (1.73–2.74)	2.50 (2.18–2.90)
ΣHREE	38.6 (28.9–43.0)	38.6 (33.5–42.2)
ΣREE	137 (88.9–163)	155 (118–178)

**Table 3:** Distribution of the elements in the factors extracted according to PCA for loadings > 0.50, loadings > 0.75 in bold, and the total variance (%) explained by each factor.

Group 1		Group 2	
Factor	Elements	Factor	Elements
Factor 1 (50.9%)	**Rb, Cr, Li, Ba, Ga, Be, V, W;** ΣHREEs, Co, Mn, Mo, Nb, Tl, Zn.	Factor 1 (81.9%)	**Er, Tm, Y, Yb, Lu, Ho, Dy;** Tb, Eu, Sm, La, Gd, Nd.
Factor 2 (23.7%)	Ta, ΣLREEs *, Ce, Hf, Zr, Bi, U; Nb, Tl.	Factor 2 (11.6%)	**Ce, Pr, Gd, Nd, Sm, La, Tb;** Eu, Dy.
Factor 3 (21.4%)	**Pb, As, Cd;** Zn, Co, Mo.		

* Sum of LREEs without Ce.

## Data Availability

Not applicable.

## Supplementary Materials

The following are available online at https://www.mdpi.com/article/10.3390/toxics10050242/s1, Figure S1. Spatial distribution of Be, Bi, Ga, Hf, Ir, Li, Nb, Rb, Ta, W, U, and Zr in sediment of Podu Iloaiei Dam Lake for July and October sampling sessions; Figure S2. Number of MRI exams performed per 1000 inhabitants in some European countries. Data compiled from OECD (2022), “Magnetic resonance imaging (MRI) exams” (indicator), https://doi.org/10.1787/1d89353f-en (accessed on 29 March 2022) and EUROSTAT, Medical technologies—examinations by medical imaging techniques (CT, MRI and PET), http://appsso.eurostat.ec.europa.eu/nui/submitViewTableAction.do (accessed on 02 May 2022); Table S1. Limit of quantification (LoQ) for inductively coupled plasma mass spectrometry determined REEs in the water and sediment samples; Table S2. The numerical evaluation of Ce and Gd anomalies and the *BSI* for the investigated sediment samples; Table S3. Principal component analysis data for TE, RE, Ce, ΣLREE*, and ΣHREE quantified in sediment samples of Podu Iloaiei Dam Lake. Bold values present loadings > 0.75; Table S4. Principal component analysis data for REE quantified in sediment samples of Podu Iloaiei Dam Lake. Bold values present loadings > 0.75.
