# Supplementary material for: Assessment of the Anthropogenic Impact and Distribution of Potentially Toxic and Rare Earth Elements in Lake Sediments from North-Eastern Romania
Source: Toxics. 2022 May 10;10(5):242. doi: 10.3390/toxics10050242 (PMC9145426; doi:10.3390/toxics10050242)
Supplement: Supplementary file 1 [file toxics-10-00242-s001.zip › toxics-1706967-supplementary.pdf]

toxics

Supplementary Materials: Assessment of the Anthropogenic Impact and Distribution of Potentially Toxic and Rare Earth Elements in Lake Sediments from North-Eastern Romania

Laurentiu Valentin Soroaga, Cornelia Amarandei, Alina Giorgiana Negru, Romeo Iulian Olariu and Cecilia Arsene

Additional information on samples treatments aspects

The water samples have undergone minimum processing, only filtration and quantitative internal standard (Indium) addition being applied. Cellulose acetate filters with a pore size of 0.2 µm (DISMIC-13CP – Advantec®) were used for all samples in order to ensure optimal retention of particulate matter that may affect the performance of ICP-MS quantification.

For the sediment samples, two processing procedures were applied: one for the estimation of water-soluble content, and the other for complete dissolution and total content analysis. For both procedures of sediment processing, special importance was placed on maintaining the total dissolved solids content (TDSC) as low as possible, as this greatly influences the performance of the analysis. However, with the use of additional aerosol dilution on the analysis step, a slightly higher TDSC may be used.

For the quantification of the water-soluble fraction, the samples were slowly dried, followed by grinding and mixing, and, eventually, weighing. Around 250 mg of sediment was used, and 25 mL of ultrapure water was added to each sample. The extraction was performed using ultrasounds for 45 minutes. Subsequently, the samples were filtered using the same 0.2 µm pore size filters, and the internal standard was added to each sample solution. The samples were stored in closed plastic containers, at room temperature, until analysis.

The water sampling was performed from the top layer, where the REEs (except Ce) will be on their lowest concentration, as they show a "nutrient-like" gradual increase of concentration with depth [13,14] and thus proved to be below the LoQ for all samples. The vertical profile for Ce is unique and opposite to the REEs group due to the fact that it shows a decrease from the high concentrations on the surface water to almost constant low concentration on the deep water. Thus, Ce should have concentrations that are above the LoQ. The fact that even Ce could not be quantified from the water samples may be related to the fact that Ce^{4+} is associated with particles and thus is removed by the filtration step, and, even more, that 35% from the total concentration could be associated with particles [13].

Element	July	October
Be	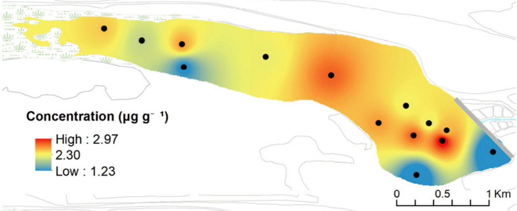	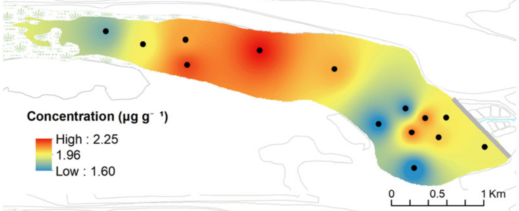
Bi	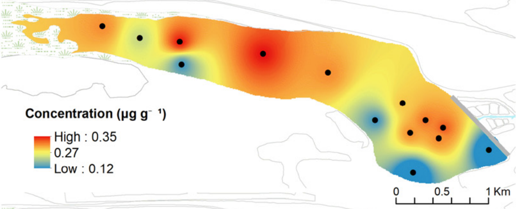	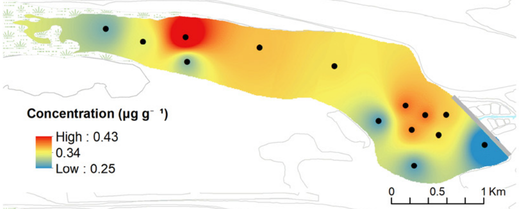
Ga	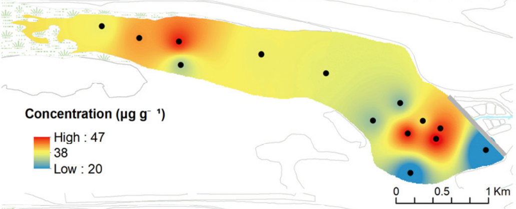	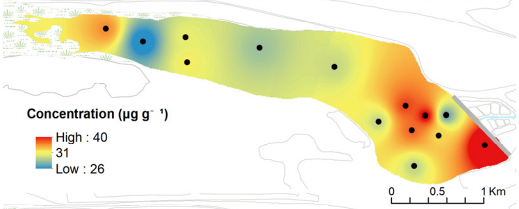
Hf	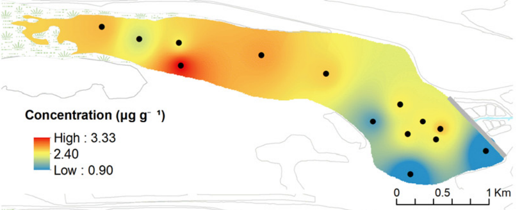	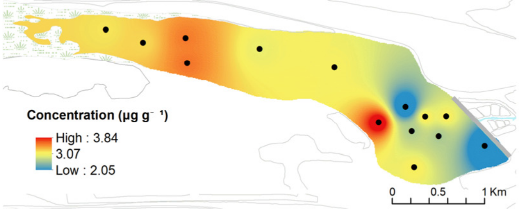
Ir	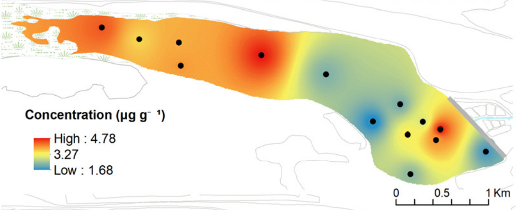	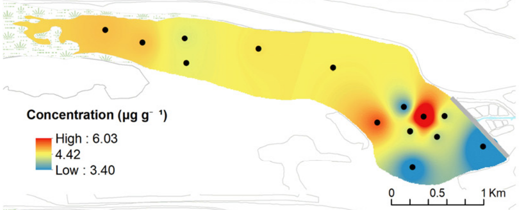
Li	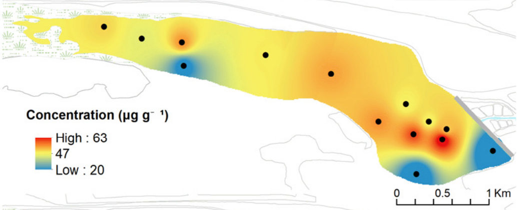	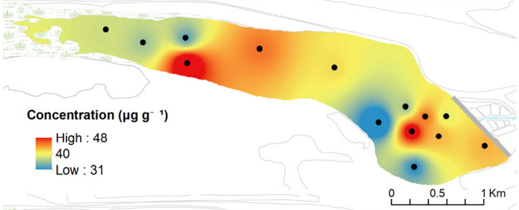
Nb	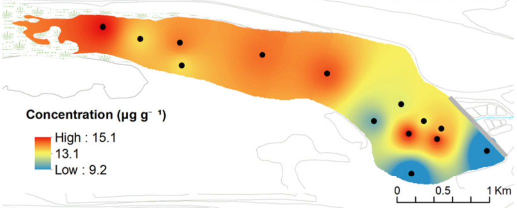	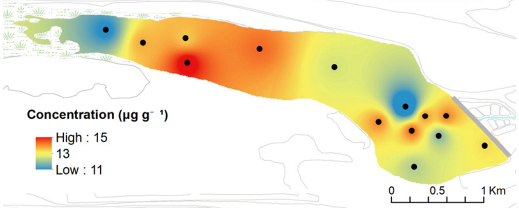
Rb	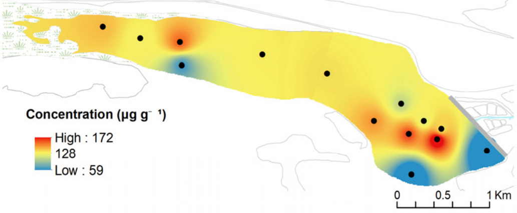	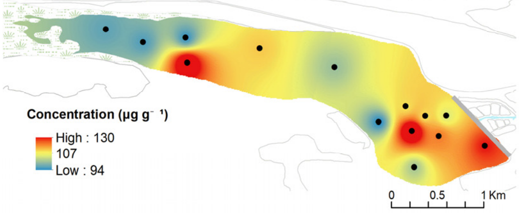

(continue)

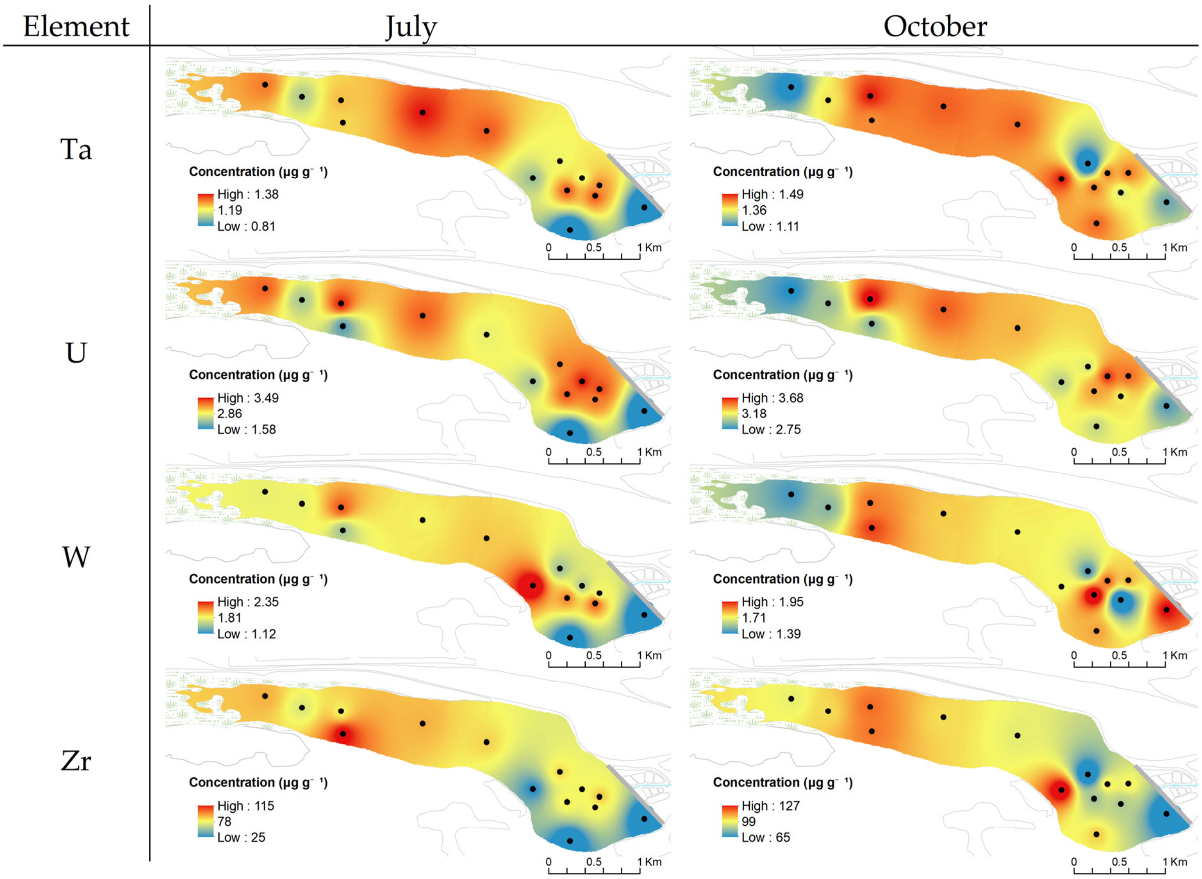

Figure S1. Spatial distribution of Be, Bi, Ga, Hf, Ir, Li, Nb, Rb, Ta, W, U, and Zr in the sediment of Podu Iloaiei Dam Lake for the July and October sampling sessions.

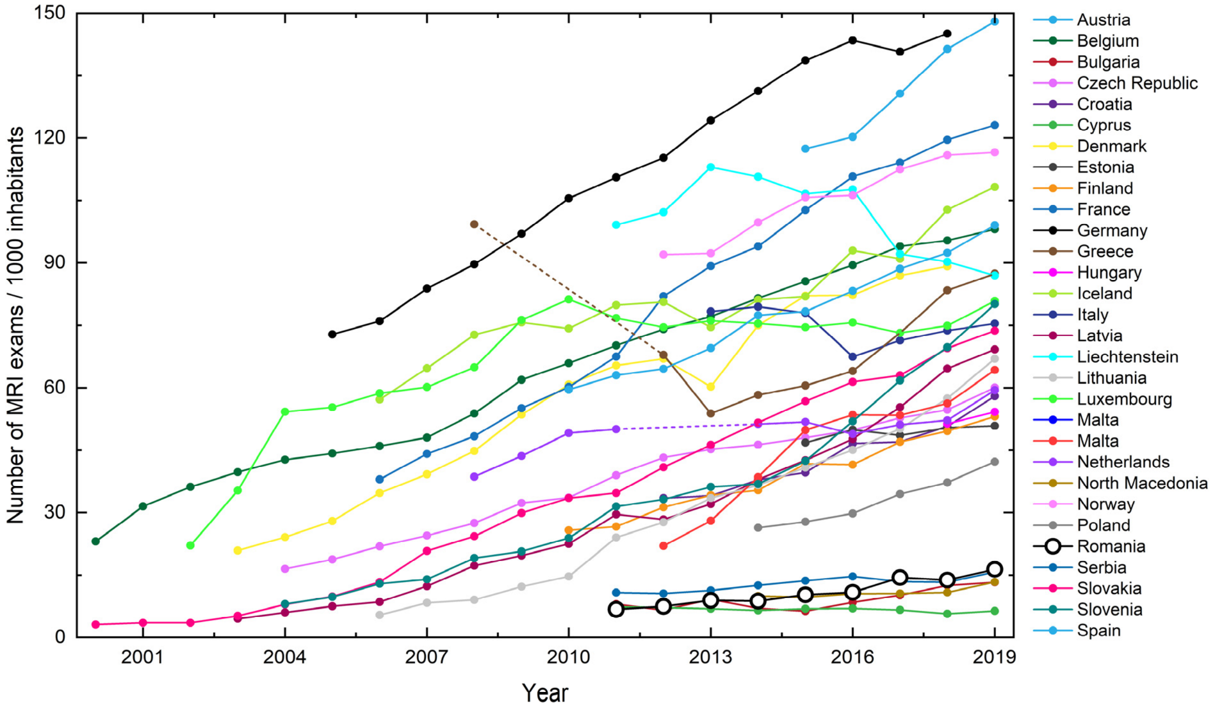

Figure S2. Number of MRI exams performed per 1000 inhabitants in some European countries. *Data compiled from OECD (2022), "Magnetic resonance imaging (MRI) exams" (indicator), https://doi.org/10.1787/1d89353f-en (accessed on 29 March 2022)*

and EUROSTAT, Medical technologies - examinations by medical imaging techniques (CT, MRI and PET), http://appsso.eurostat.ec.europa.eu/nui/submitViewTableAction.do (accessed on 02 May 2022).

Table S1. Limit of quantification (LoQ) for inductively coupled plasma mass spectrometry determined REEs in the water and sediment samples.

Element	Water	Sediment water soluble	Sediment total content
	LoQ (μg L^{-1})	LoQ (μg g^{-1})	LoQ (μg g^{-1})
^{89}Y	0.316	0.006	0.653
^{139}La	0.167	0.004	0.649
^{140}Ce	0.174	0.012	0.831
^{141}Pr	0.174	0.020	0.099
^{146}Nd	0.186	0.035	0.562
^{147}Sm	0.076	0.024	0.122
^{153}Eu	0.143	0.017	0.083
^{157}Gd	0.386	0.016	0.089
^{159}Tb	0.054	0.008	0.040
^{163}Dy	0.270	0.010	0.051
^{165}Ho	0.056	0.011	0.056
^{166}Er	0.149	0.013	0.067
^{169}Tm	0.158	0.008	0.040
^{172}Yb	0.281	0.015	0.075
^{175}Lu	0.027	0.005	0.023

Table S2. The numerical evaluation of Ce and Gd anomalies and BSI for the investigated sediment samples.

	July			October		
	Ce/Ce*	Gd/Gd*	BSI	Ce/Ce*	Gd/Gd*	BSI
S-1	0.62	1.20	1.25	0.67	1.19	1.26
S-2	0.42	1.19	1.24	0.92	1.26	1.28
S-3	0.49	1.22	1.24	0.81	1.20	1.24
S-4	0.56	1.21	1.21	0.75	1.23	1.23
S-5	0.78	1.23	1.25	0.91	1.28	1.28
S-6	0.73	1.23	1.26	0.88	1.20	1.27
S-7	0.33	1.16	1.21	0.85	1.24	1.19
S-8	0.64	1.21	1.26	0.49	1.20	1.25
S-9	0.49	1.19	1.26	0.78	1.21	1.24
S-10	0.65	1.24	1.26	0.91	1.20	1.26
S-11	0.49	1.22	1.25	0.78	1.25	1.27
S-12	0.69	1.21	1.27	0.91	1.22	1.26
S-13	0.39	1.15	1.21	0.71	1.18	1.23
S-14	0.47	1.16	1.28	0.37	1.20	1.21
Minimum	**0.33**	**1.15**	**1.21**	**0.37**	**1.18**	**1.19**
Average	**0.55**	**1.20**	**1.25**	**0.77**	**1.22**	**1.25**
Maximum	**0.78**	**1.24**	**1.28**	**0.92**	**1.28**	**1.28**

Ce/Ce* - Cerium anomaly; Gd/Gd* - Gadolinium anomaly; BSI – Bell Shaped Index.

Table S3. Principal component analysis data for TE, RE, Ce, ΣLREE*, and ΣHREE quantified in sediment samples of Podu Iloaiei Dam Lake. Bold values present loadings > 0.75.

Element	Factor 1	Factor 2	Factor 3
Li	**0.92**	0.18	0.16
Be	**0.87**	0.23	0.13
Mn	0.69	−0.46	0.32
Co	0.69	0.06	0.62
Zn	0.59	0.23	0.63
Ga	**0.89**	−0.04	0.27
As	−0.19	−0.09	**0.78**
Rb	**0.97**	0.10	0.11
Zr	0.00	**0.91**	0.02
Cd	0.47	0.18	**0.78**
Ba	**0.90**	−0.22	−0.12
Tl	0.60	0.66	0.37
Pb	0.05	0.32	**0.78**
Bi	0.20	**0.76**	0.55
U	0.39	**0.75**	0.40
V	**0.84**	0.29	0.37
Cr	**0.92**	0.19	0.19
Nb	0.64	0.71	0.02
Mo	0.64	−0.28	0.56
Hf	−0.02	**0.92**	0.01
Ta	0.19	**0.95**	0.04
W	**0.81**	0.29	−0.15
Ce	−0.12	**0.93**	0.07
ΣLREE*	0.17	**0.95**	0.05
ΣHREE	0.72	0.49	−0.15
Eigenvalue	**12.7**	**5.9**	**2.8**
Total variance %	**50.9**	**23.7**	**11.0**
Cumulative variance %	**50.9**	**74.6**	**85.6**

LREE*-LREE without Ce.

Table S4. Principal component analysis data for REE quantified in sediment samples of Podu Iloaiei Dam Lake. Bold values present loadings > 0.75.

Element	Factor 1	Factor 2
La	0.54	**0.80**
Ce	−0.07	**0.90**
Pr	0.44	**0.89**
Nd	0.52	**0.84**
Sm	0.55	**0.81**
Eu	0.58	0.74
Y	**0.90**	0.18
Gd	0.53	**0.84**
Tb	0.62	**0.76**
Dy	**0.84**	0.52
Ho	**0.86**	0.47
Er	**0.94**	0.31
Tm	**0.93**	0.27
Yb	**0.88**	0.39
Lu	**0.86**	0.42
Eigenvalue	**12.3**	**1.7**
Total variance %	**81.9**	**11.6**
Cumulative variance %	**81.9**	**93.5**
